# Hygiene

**Published:** 1836-01

**Authors:** 


### HYGIENE.

### *On the Diseases of Printers.* By A. Chevallier.

[M. Chevallier has been struck with the incomplete way in which the diseases of artisans have been treated, and with the exaggerations and false facts with which works on this subject abound. In the elaborate memoir now before us, he has endeavoured to investigate the diseases of one class, those of printers; and the analysis which we shall make will give an idea of the best plan of pursuing the investigation of these diseases, as well as an exposure of the loose way in which statements have been made by others. In order to collect information, the physician (says M. Chevallier) must study the subject in the workshop and manufactory. He must consult the oldest and most clever workmen in many places; make notes of the information thus obtained, and verify it by examining other workmen on the facts; compare this information with that of the masters; consult practitioners who treat these diseases; and establish statistics of mortality.]

Both workmen and masters have been examined, and some notion of the difficulty of the work may be had, from the fact that M. Chevallier received only 33 written answers to 400 letters to masters.

Is a sedentary life a source of disease?—Printers are less sedentary than the majority of workmen.

Do they become short-sighted?—The work of a compositor does not cause myopia, but increases it. The sight of a compositor is fatigued by night-work, by reading proofs, by correction on the form,* by small type, by the brilliancy of new type, by the difficulty of reading some manuscripts; and by this constant fatigue, the sense of sight is impaired and destroyed. Many compositors at 45 take to glasses. Excess of drink aids the most in weakening sight.

Do they become amaurotic?—Only one case was known of amaurosis, and there are between 3000 and 4000 printers in Paris, two thirds of whom are compositors.

Are their nights disturbed by dreams of types and presses?†—The young and ardent sometimes dream, but it does not fatigue them, and it wears off.

Are they subject to swollen legs, varices, and ulcers?—The habit among compositors of standing at their work causes, in some, trembling, fatigue, and swelling of the legs; and when they become old, and drink hard, varices and ulcers follow.‡ The pressmen, who incessantly work on foot, and who walk in this manner a distance equal to seven or eight leagues daily, are more particularly subject to the same affection. These accidents are ameliorated by sitting during composing, which may be done, except in printing newspapers, where great activity is required; and by wearing laced boots.

Are they subject to colica pictonum?—In some printing-houses it is common, and in others unknown. Those that assert its frequency allow that it has diminished principally, 1st, from a change in the metal of which the types are made; 2d, from precautions taken to clean the boxes and types from powder and dust; 3d, from the greater cleanliness of the workmen, who generally wash their hands before eating and as they leave work. The attacks are not severe; they affect, 1st, apprentices, who put the types in their mouth, or who clean the boxes; 2d, compositors who, whilst making their corrections over the form, put the types in their mouth. These accidents are most frequent when the types are new; the layer of printing ink upon which, hinders the saliva from acting on the metal; for cholic in printers seems to depend on the introduction into the economy of some of the metal, or metallic oxide, which is detached from the types. Some expose themselves to all these causes without suffering: some elderly men were pointed out who never washed their hands, and never had cholic.

Do wounds made during work heal with facility?—Wounds heal readily, but chaps do not, owing to the solution of potash in which the forms are washed. Accidents occasionally happen, and it is therefore necessary when a wound is made, that the part should be kept very clean, and if the case is bad, work should be suspended.

Are compositors subject to tics [neuralgia—spasms] of the hands and face?—Many contract odd movements and grimaces whilst at work, but these are not diseases, and may be corrected.

Are they subject to inflammatory diseases?—Careful inquiry shows that they are not common; and particularly so, when it is recollected that the men take little care in passing from hot to cold places; that they drink hard, and occasionally work excessively hard after loss of time, or when their work presses.

Are they subject to diseases of the chest?—It should be remembered, that from the work of a compositor being gentle and sedentary, as well as somewhat instructive and liberal, it is often chosen by weakly young men, born in the city,

* "Form," which is frequently mentioned, is the technical term for types arranged so as to be fit for taking off an impression.

[† This certainly appears a very trifling interrogatory.—Ed.]

‡ We believe, from some inquiries of our own, made in London, that these affections of the lower extremities of printers, are by no means confined to the advanced in age, or the intemperate.—Ed.]

who bring with them certain dispositions to disease. It does not appear that printers are more subject to diseases of the chest than others, and such affections proceed from several causes; such as, some cause external to the printing-house; the bent position in which some compositors work; and excess of work, or dissipation.

Are they subject to hernia?—The pressmen are subject to it; but the compositors more rarely. It is a consequence of imprudence and want of precaution. It may arise from the efforts of pressmen to carry, raise, and put on the press, forms, weighing from 40 to 60 pounds, which they usually carry in one hand; or from attempts of compositors to put the form on the table, in order to make corrections.

Are they subject to rupture of the muscles?—No: but the pressmen are subject to an affection (called by them *rossignol),* which they regard as a painful lassitude, or rather a sprain, followed by swelling of the radio-carpal joint. It comes on, 1st, in the workmen, who ink the forms, when they take again to their work after some absence; 2d, in pressmen who have given up their labours, and who attribute it to turning a handle which brings the form under that part of the machine which makes the impression; 3d, when the strap connected with the cylinder which rolls the press happens to break, and the shock falls on the muscles of the wrist. It is not serious, and is cured by a few days' rest. It is less frequent in those printing-houses where the balls used to ink the forms have been replaced by rollers; as it was necessary, in order to cover the former with ink, to turn the two together in a contrary direction, requiring a motion very fatiguing to the fore-arm.

Are they subject to dropsy?—No.

Are they subject to paralysis?—Some cases of paralysis have happened among the old, or debauched, or after falls.

Are they subject to aneurisms, or diseases of the heart?—The evidence of both masters and men was in the negative. The work of the press, which requires repeated efforts of the muscles of the arms, chest, and pelvis, exposes workmen more than others to these diseases; but they will become less frequent in proportion as the American and Stanhope presses are more used, which require less personal exertion.

Are they subject to scrofula, and œdema?—No: a great many lose their colour, although their flesh is firm, and particularly among the compositors of newspapers.

To what diseases are they subject?—According to their masters, the printers who are sober, and regular, are not subject to any disease arising directly from their calling. The greater part of their diseases arise from negligence, carelessness, and excesses.

Longevity of printers. Cadet de Gassicourt has stated, that printers seldom live beyond 45 years; and according to Thackrah, they rarely pass 50. This is not the case; for in 23 printing-houses in Paris, the age of the workmen was respectively as follows:—

| NO. | | NO. | |
|---|---|---|---|
| 1 | from 18 to 60 years | 13 | from 40 to 50 years |
| 2 | .... 15 to 50 | 14 | .... 30 to 60 |
| 3 | .... 12 to 75 | 15 | .... 10 to 70 |
| 4 | .... 18 to 55 | 16 | .... 25 to 50 |
| 5 | .... 18 to 50 | 17 | .... 20 to 60 (2) |
| 6 | .... 18 to 50 | 18 | .... 20 to 60 |
| 7 | .... 18 to 60 | 19 | .... 17 to 50 |
| 8 | .... 20 to 50 | 20 | .... 20 to 45 |
| 9 | .... 20 to 50 | 21 | .... 18 to 65 |
| 10 | .... 18 to 40 | 22 | .... 15 to 70 (3) |
| 11 | .... 20 to 55 | 23 | .... 17 to 60 |
| 12 | .... 20 to 40 | | |

M. Chevallier was informed by a workman, that eighteen years ago there was one printing-house in which forty men were employed, twenty-five of whom were between 50 and 70 years: so that it was nicknamed the grey-beard printing-house. The names are given of seventeen printers, now working in Paris, fourteen of whom are 70 years old, one 68, one 62, and one 80. A man named Dubois worked for Didot, jun. until 86 years old, when he died; and another worked until 83 or 84 years old. The tables of mortality of the hospitals in 1831, enumerate twenty-five printers who died between 55 and 78 years. In the hospital for the old, four died at the ages of 69, 78, 64, and 75. M. Chevalier endeavoured to ascertain from statistical tables, the mean duration of the life of printers, and the diseases of which they died during ten years; but so little care had been taken in specifying the branches of the trade which they pursued, that such an undertaking became impossible.

M. Chevallier terminated his inquiry by the question, By what means could the condition of printers be ameliorated?—It was the opinion of most, that this could only be done by endeavouring to convince them of the evils arising from drink, and other excesses, and of the necessity of regular habits, of economy, and of putting by a part of their wages. Some good might be effected by the masters following the example of M. Beauvisage, a dyer, and giving gold medals annually to the best, most sober, and economical workmen. The masters might also exert considerable influence over the men, by only employing sober workmen; and by persuading them to put by a small sum on each pay-day, as a reserve in case of illness or age.

The master-printers should, for the interest of the public health, and that of their workmen: 1. Recommend the men spectacles early enough to preserve their sight. 2. Avoid night-work; and use lamps instead of candles, which give more light, and are less unwholesome. 3. Ventilate the shops, especially when they are being cleaned. 4. Give the compositors seats. 5. Exercise regularity among the pressmen, forbidding them, after debauches, to do six days' work in three: and require that those who work together should be of equal age and strength, so that one should not be over-worked by keeping up with his companion. 6. Recommend laced boots to those whose legs swell. 7. Point out to apprentices and compositors the bad effects of holding type in the mouth. 8. Recommend the men to wash their hands with alkaline water on leaving their work, and before meals. 9. Dress wounds carefully, and keep them clean. 10. Advise warm clothing on leaving the workshop when heated. 11. Explain the dangers of excess. 12. Keep up a moderate temperature in the workshops. 13. Prefer sober workmen. 14. Establish workshops in large, airy, dry places; and the printed paper should not be dried in them; these sheets in drying injure the workmen, by causing a humid atmosphere. The workmen in order to preserve their health should, 1. Ventilate their workshops, by opening the windows at night, and having a sufficient supply of fresh air during the day to replace the air vitiated by respiration, emanations from the body and candles. 2. Rest for some time, if the eyes are weary, or if there is lassitude, fatigue, &c. 3. Guard against bad habits, such as are called in printing-houses tics, which are difficult to lose. 4. Work with moderation, for hard labour like the press, carried to an extreme, debilitates the workman, so that he is unable to work as he advances in years. 5. Preserve moderate warmth by suitable clothing, and keep the feet warm by proper shoes. 6. Live temperately and regularly, so as not to be obliged, from a debauch of several days, to work excessively and live on insufficient food. On days of rest from work walk in the open air, exercise the muscles with gymnastic games, such as ball, running, and long walks.

[To the foregoing important observations, we would merely add, that *compositors* will consult their health by sleeping at a short distance from the towns where they work, and preserving, while at work, the upright position. The occupation of *pressmen* is on the whole healthy; but each should bear in mind the danger of rupture and other mechanical lesions, by suddenly lifting heavy weights of type. The present notice will meet the eye of so many readers interested in whatever relates to literature, or to those in any way connected with it, that we avail ourselves of the opportunity of mentioning that there exists in London, a Society called the "Printer's Pension Society," the assistance of which, by donations or small annual subscriptions from the affluent and liberal, may be the means of preserving many an industrious printer, who has done *his* part in presenting reading to the public, and thus contributing to furnish instruction and amusement, or the best solace of many minds, to thousands of readers, from distress in sickness, or poverty in age.]

*Annales d'Hygiène publique, Avril,* 1835.

